# Atypical Color Preference in Children with Autism Spectrum Disorder

**DOI:** 10.3389/fpsyg.2016.01976

**Published:** 2016-12-23

**Authors:** Marine Grandgeorge, Nobuo Masataka

**Affiliations:** ^1^Université de Rennes 1, UMR CNRS 6552Rennes, France; ^2^Primate Research Institute, Kyoto UniversityInuyama, Japan

**Keywords:** color preference, autism spectrum disorder, hyper-sensation, vision

## Abstract

So far, virtually no study has ever investigated color preference in children with autism spectrum disorder (ASD). In order to address this issue, 29 boys with ASD varying in age between 4 and 17 years, and 38 age-matched typically developing (TD) boys were studied regarding their preference among six colors: red, pink, yellow, brown, green, and blue, in clinical settings. When mean rank of preference was computed in each of the ASD and TD groups with regard to each color, it was found that boys with ASD were significantly less likely than TD boys to prefer yellow and more likely than TD boys to prefer green and brown colors. These results appear to be caused by hyper-sensation characteristic of ASD, due to which boys with this disorder perceive yellow as being sensory-overloading.

## Introduction

Studies of children’s color preference have a long history. Virtually, all of them have investigated typically developing (TD) children. Pioneering studies concerning this issue (Bornstein, 1975; Zentner, 2001) as well as a relatively recent (but the best-known) study (Franklin et al., 2008a) reported that preschool-aged boys and girls prefer red to all other colors. A similar preference for red has been reported for infants (Franklin et al., 2010). Although other studies have presented evidence for a preference for blue in newborns (Teller et al., 2005; Zemach et al., 2007), there is certainly a general consensus that TD children have a preference for primary colors (such as red and blue) rather than secondary colors (such as pink and orange). As the possible functional significance of such color-preference, moreover, the need to discriminate subtle changes in skin color of other persons due to their emotional states (e.g., an angry face is reddish and a sad face is blue) has been argued (Changizi et al., 2006). Such reasoning apparently assumes that the preference of TD children for primary colors is a predispositional one.

Concerning children with autism spectrum disorder (ASD), neurodevelopmental disorders with unusual sensory processing, some anecdotal evidence from parents, caretakers, teachers of persons with ASD and persons with ASD themselves suggests that children with this disorder may perceive color differently to TD children (Franklin et al., 2008b). Especially, color obsession with green has been abundantly documented (Higashida, 2013; Silberman, 2015; Masataka, in press). In one case, for instance, an 11-year-old boy with ASD continued to use a green straw for the purpose of stimming for more than 3 years (Silberman, 2015). Seemingly odd color perception has also been reported in experimental studies with children with ASD. For example, Brian et al. (2003) unexpectedly found a facilitating effect by colored stimuli when investigating inhibitory mechanisms in participants with ASD, while such effect was not observed in neurotypical controls. The authors argued that in ASD, ‘stimulus features such as color may be encoded too readily, and thus are detected more easily than is typically the case.’ Subsequently, a similar effect was found with respect to the cueing task, where invalid color cues resulted in greater costs for participants with ASD than for neurotypical controls (Greenway and Plaisted, 2005). Those studies consistently found enhancement of task performance by colored materials in those children.

Other studies (Ludlow et al., 2006, 2008, 2012) found, in clinical settings, a perceptual benefit from the use of colored filters in a large proportion of individuals with ASD. The overlays were designed to sample chromaticity systematically and comprehensively so that if there was any color that was beneficial, there was an available overlay or combination of overlays providing a close approximation to this color. Another study, on the other hand, attempted to compare the categorical perception of color between children with ASD and TD children (Franklin et al., 2008b) and reported that the strength of categorical perception of color did not differ between ASD and TD children.

Taken together, the above findings have led us to hypothesize that while the basic mechanism underlying perceptual categorization of colors would not differ between people with ASD and without ASD, the enhanced sensitivity to sensory stimulation in general that is characteristic of ASD (Markram and Markram, 2010) would influence color perception exhibited by people with this disorder, and this would result in aversion to some specific colors that are usually favored by neurotypical people. The current study was designed to explore this possibility, using the same stimuli as those in the previous pioneering work, on the assumption that ASD children possess perceptual color categories equivalent to those in TD children.

## Materials and Methods

This investigation was conducted according to the principles expressed in the Declaration of Helsinki. All experimental protocols were consistent with the Guide for Experimentation with Humans, and were approved by the Institutional Ethics Committee, of the Primate Research Institute, Kyoto University (#2011-150). The authors obtained written informed consent from parents of all participants involved in the study.

### Participants

A group of 29 children with ASD aged 4 to 17 years (*M* = 8.8; *SD* = 3.0) and 38 TD children aged 4 to 17 years (*M* = 9.8; *SD* = 4.0) were studied in the current study. They were all males. There was no significant difference between the mean age of each participant group [*t*(65) = 1.15, *p* = 0.25]. All participants were French, right-handed, naïve as to the purpose of this study, and had normal or corrected-to-normal vision. They did not have any difficulty in color sensing.

Twenty-nine children with ASD were recruited for the current study. Based on direct clinical observation of each child by an independent child psychiatrist, a diagnosis of autism was made according to ICD-10 (World Health Organization, 1994) as well as DSM-IV (American Psychiatric Association, 1994). On the basis of such criteria, each participant in the group of children with ASD were diagnosed as either F84.0, F84.9, or F84.8. Moreover, such diagnoses were also confirmed by the Autism Diagnostic Interview-Revised (ADI-R), an extensive, semi-structured parental interview (Lord et al., 1994) that was conducted by an independent psychiatrist. The ADI-R provides information about the presence of verbal language skills, defined as daily, functional and comprehensive use of spontaneous phrases of at least three words and occasionally a verb. All of the participant ASD children were found to express verbal language. All of the TD children were recruited via the board of education in a small city in France. All of them attended normal classes corresponding to their chronological age level. None of the participants included in the groups of TD children met any diagnostic criterion for autism or any other pervasive developmental disorder.

In order to examine a possible developmental change of color preference, each of the ASD children and the TD children was classified into one of three age groups: one age group consisted of children aged 4 to 7 years (9 ASD children and 13 TD children), one group consisted of children aged 8 to 10 years (9 ASD children and 17 TD children), and the remaining group consisted of children aged 11 to 17 years (11 ASD children and 8 TD children). Given that the ANOVA (analysis of variance) assumptions are certainly met, such division of the entire participant group should be coherent to investigate possible changes within a developmental perspective.

### Procedure

The materials used in the current study consisted of six 35 cm × 50 cm cardboard rectangles colored red, yellow, pink, blue, green, or brown. They were essentially the same as the materials used in the previous study (Zentner, 2001) so that we could compare our results obtained here with those reported there. Hue, luminance, and chroma of each color stimulus that was specified according the Munsell system of color notation were as follows: red, 7.5R, 4, 14; yellow, 10Y, 8.5, 12; pink, 7.5PR, 6, 10; green, 2.5G, 3, 8; blue, 10B, 7, 8; brown, 10R, 3, 10.

Protocol of testing was also the same as that in the previous study (Zentner, 2001). Participants were tested individually in a quiet room under daylight conditions. All of the six cardboards were presented to the participant, who was seated on a chair and asked to pick the color he liked. Their preference rank among the cardboards was measured by forced-choice paired-comparison procedure. Each time, the participant picked the color, a preference for that color was recorded. For the statistical analysis, the preference score was computed for the color by subtracting its preference rank from the number of the stimulus color (6).

## Results

**FIGURE 1:**
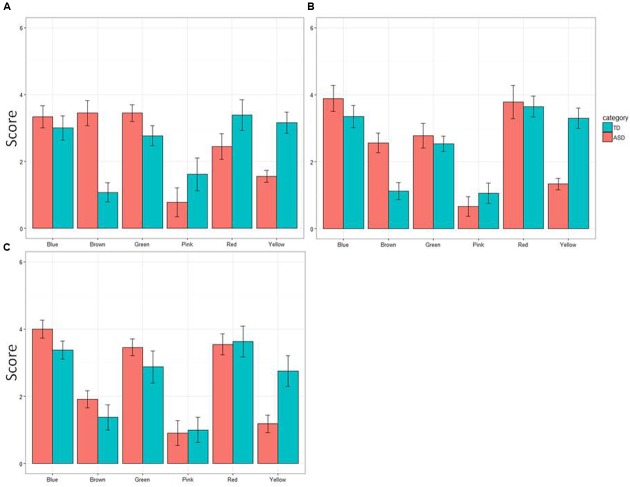
**Mean preference scores (error bars: SDs) of six colors in children with autism spectrum disorder (ASD) and in typically developing (TD) children. (A)** 4- to 7-year-olds, **(B)** 8- to 10-year-olds, and **(C)** 11- to 17-year-olds.

The overall results of the experiment are summarized in **Figure 1**, which shows the overall mean rank of the six colors in the group of TD children and that of ASD children. When the collected data were analyzed using a 2 (ASD/TD, PARTICIPANT) × 3 (age groups, AGE) ANOVA for each of the six colors, one of the two main effects (PARTICIPANT) was statistically significant for yellow, *F*(1,61) = 49.60, *p* = 0.000, $\eta_{p}^{2}$ = 0.284 and for green, *F*(1,61) = 5.03, *p* = 0.029, $\eta_{p}^{2}$ = 0.114. The another main effect (AGE) was significant neither for yellow, *F*(2,61) = 0.84, *p* = 0.44, $\eta_{p}^{2}$ = 0.028, nor green, *F*(2,61) = 1.50, *p* = 0.23, $\eta_{p}^{2}$ = 0.53. The interaction between PARTICIPANT and AGE was not significant for yellow, *F*(2,61) = 0.25, *p* = 0.78, $\eta_{p}^{2}$ = 0.08, or for green, *F*(2,61) = 0.28, *p* = 0.76, $\eta_{p}^{2}$ = 0.09, either.

For the color brown, both the main effect, PARTICIPANT and the interaction between PARTICIPANT and AGE were significant, *F*(1,61) = 33.06, *p* = 0.0000, $\eta_{p}^{2}$ = 0.35 for PARTICIPANT, and *F*(2,61) = 4.11, *p* = 0.021, $\eta_{p}^{2}$ = 0.119 for PARTICIPANT × AGE. However, the another main effect was not significant, *F*(1,61) = 1.89, *p* = 0.16, $\eta_{p}^{2}$ = 0.062. Subsequent analyses of simple main effects (Bonferroni correction) revealed that the mean rank of preference for brown was smaller in 11- to 17-year-old children with ASD than 4- to 7-year-old children with ASD, *p* = 0.001, as well as 8- to 10-year-old children with ASD, *p* = 0.03. The mean rank of preference of 4- to 7-year-old children with ASD did not differ from that of 11- to 17-year-old children with ASD, *p* = 0.31.

In contrast, neither of the two main effects nor the interaction between them was significant for red, *F*(1,61) = 0.70, *p* = 0.41, $\eta_{p}^{2}$ = 0.012 for PARTICIPANT, *F*(2,61) = 1.77, *p* = 0.18, $\eta_{p}^{2}$ = 0.068 for AGE, *F*(2,61) = 0.98, *p* = 0.38, $\eta_{p}^{2}$ = 0.081 for PARTICIPANT × AGE, for blue, *F*(1,61) = 3.39, *p* = 0.08, $\eta_{p}^{2}$ = 0.046 for PARTICIPANT, *F*(2,61) = 1.25, *p* = 0.29, $\eta_{p}^{2}$ = 0.040 for AGE, *F*(2,61) = 0.09, *p* = 0.91, $\eta_{p}^{2}$ = 0.003 for PARTICIPANT × AGE, and for pink, *F*(1,61) = 1.90, *p* = 0.17, $\eta_{p}^{2}$ = 0.028 for PARTICIPANT, *F*(2,61) = 0.51, *p* = 0.61, $\eta_{p}^{2}$ = 0.040 for AGE, *F*(2,61) = 0.41, *p* = 0.66, $\eta_{p}^{2}$ = 0.003 for PARTICIPANT × AGE.

## Discussion

Regarding TD children, the results of the current study are consistent with those reported previously (Zentner, 2001; Franklin et al., 2010). Red was the most preferred color. Blue was close to it, and then yellow followed. The least preferred color was brown. As reported in a recent study, pink was also avoided by boys (LoBue and DeLoache, 2011). Such findings were also confirmed in children with ASD. However, their preference score for yellow was low, and that for green as well as that for brown was conversely elevated.

Since the presented color categories used here were restricted, it appears difficult to draw any definite conclusion from these results. Given the relatively small sample size in each of the three age groups, the failure to find any difference in preference scores between TD children and children with ASD with regard to red, blue and pink might be attributable to a ceiling/floor effect. Apart from this issue, however, the fact should be noted that children with ASD were certainly likely to avoid yellow and, conversely, to favor green and brown. These findings are certainly those predicted by our hypothesis outlined above. Moreover, their preference for green is consistent with anecdotal evidence that has been reported so far (Higashida, 2013; Silberman, 2015; Masataka, in press).

In order to explain these results, the fact that the yellow color had the highest luminance value among the colors tested should not dismissed. The observed aversion to this color might reflect hyper-sensitivity of children with ASD to luminance. There is also a general consensus that yellow is the most fatiguing color (Kernell, 2016). It is well known that our eyes are provided with three different types of cone cells for color perception, L, M, and S, which correspond to the perception of red, green, and blue light, respectively. When yellow is perceived, however, both L and M must be involved. The perception of yellow should thus be the most heavily sensory-loaded of the perception of any type of color. Its perception is bearable for TD children, but could be over-loaded for children with ASD whose sensitivity to sensory stimulation is enhanced.

It is often reported that children with ASD are hyper-sensitive to tactile, auditory and visual input. In the auditory domain, they exhibit enhanced discrimination between auditory stimuli, more accurate local target detection of auditory stimuli, and diminished global interference with auditory processing (Takahashi et al., 2014). In the visual domain, they exhibit enhanced visual discrimination capabilities, faster target detection in feature and conjunctive visuals searches, more accurate local target detection, etc (Markram and Markram, 2010). The current study suggests the possibility that such a phenomenon also occurs in the domain of color perception. The color yellow as a sensory stimulus, which is normal to TD children, may be difficult to bear for children with ASD.

Recently, hyper-sensation as well as hyper-attention characteristic of ASD has been neurologically explained in terms of an underlying neural underconnectivity among cortical areas in this disorder (Just et al., 2004), which could negatively impact or slow integration or communication among cortical regions involved in visual imagery processing as well as language. This explanation attributes many of the widespread abnormalities in psychological functioning in ASD to an impairment in the coordination and communication between key brain processing centers. One of the main predictions made based on this explanation is that any facet of psychological and neurological function that is dependent on the coordination or integration of brain regions is susceptible to disruption in ASD. Neurologically, a generally accepted basic account of color processing holds that color vision starts in the retina, that then, parvocellular and koniocellular cells in the lateral geniculate nucleus code for chromaticity, and magnocellular cells for luminance, thus providing different pathways to the visual cortex where various color-selective neurons are found (Kernell, 2016). The pattern of results in the current study could arise from disruption of one or more of these different biological and neurological processes. Further studies are needed to explore this.

A person who is suffering from sensory overload will naturally avoid such an overly strong stimulus as aversive. Such avoidance could manifest itself as the observed atypical color preference in the current study. The fact that the robust avoidance of brown was observed only in children with ASD under 11 years might suggest the possibility that hyper-sensitivity is most intense during this developmental period in this disorder. Apparently this is also the issue to be investigated in the near future.

## Author Contributions

NM designed the study. MG collected the data. NM analyzed the data and drafted the manuscript. Both read the draft and approved it.

## Conflict of Interest Statement

The authors declare that the research was conducted in the absence of any commercial or financial relationships that could be construed as a potential conflict of interest.
